# Direct interplay between stereochemistry and conformational preferences in aminoacylated oligoribonucleotides

**DOI:** 10.1093/nar/gkz902

**Published:** 2019-10-15

**Authors:** Anton A Polyansky, Mathias Kreuter, John D Sutherland, Bojan Zagrovic

**Affiliations:** 1 Department of Structural and Computational Biology, Max Perutz Labs, University of Vienna, Campus Vienna Biocenter 5, Vienna A-1030, Austria; 2 National Research University Higher School of Economics, Moscow 101000, Russia; 3 MRC Laboratory of Molecular Biology, Francis Crick Avenue, Cambridge Biomedical Campus, Cambridge CB2 0QH, UK

## Abstract

To address the structural and dynamical consequences of amino-acid attachment at 2′- or 3′-hydroxyls of the terminal ribose in oligoribonucleotides, we have performed an extensive set of molecular dynamics simulations of model aminoacylated RNA trinucleotides. Our simulations suggest that 3′-modified trinucleotides exhibit higher solvent exposure of the aminoacylester bond and may be more susceptible to hydrolysis than their 2′ counterparts. Moreover, we observe an invariant adoption of well-defined collapsed and extended conformations for both stereoisomers. We show that the average conformational preferences of aminoacylated trinucleotides are determined by their nucleotide composition and are fine-tuned by amino-acid attachment. Conversely, solvent exposure of the aminoacylester bond depends on the attachment site, the nature of attached amino acid and the strength of its interactions with the bases. Importantly, aminoacylated CCA trinucleotides display a systematically higher solvent exposure of the aminoacylester bond and a weaker dependence of such exposure on sidechain interactions than other trinucleotides. These features could facilitate hydrolytic release of the amino acid, especially for 3′ attachment, and may have contributed to CCA becoming the universal acceptor triplet in tRNAs. Our results provide novel atomistic details about fundamental aspects of biological translation and furnish clues about its primordial origins.

## INTRODUCTION

As a unique combination of functional groups, the 2′-3′ *cis*-diol in the 3′-terminal adenosine of tRNA (A76) directly affects different aspects of biological translation. Specifically, the two neighboring 2′- and 3′-hydroxyl groups in the A76 ribose are critically involved in tRNA charging, proofreading and accommodation as well as ribosomal protein synthesis (1–3). Importantly, covalent linkage of amino acids to tRNA by aminoacyl-tRNA synthetases (tRNA charging) occurs at either 2′ or 3′ positions of the A76 ribose, with the two stereoisomers interconverting via transacylation and the charging kinetics depending on the non-connective hydroxyl (3–6). In addition, formation of the aminoacyl adenylate intermediate in tRNA charging hinges upon the presence of the 2′-hydroxyl (3,7,8), while replacing the non-connective hydroxyl with a proton dramatically impacts the subsequent proofreading of charged tRNAs by aminoacyl-tRNA synthetases (3,9–11). Despite such direct involvement of the two hydroxyls at different stages of translation, our understanding of the underlying microscopic mechanisms is largely incomplete.

**Figure 1.**
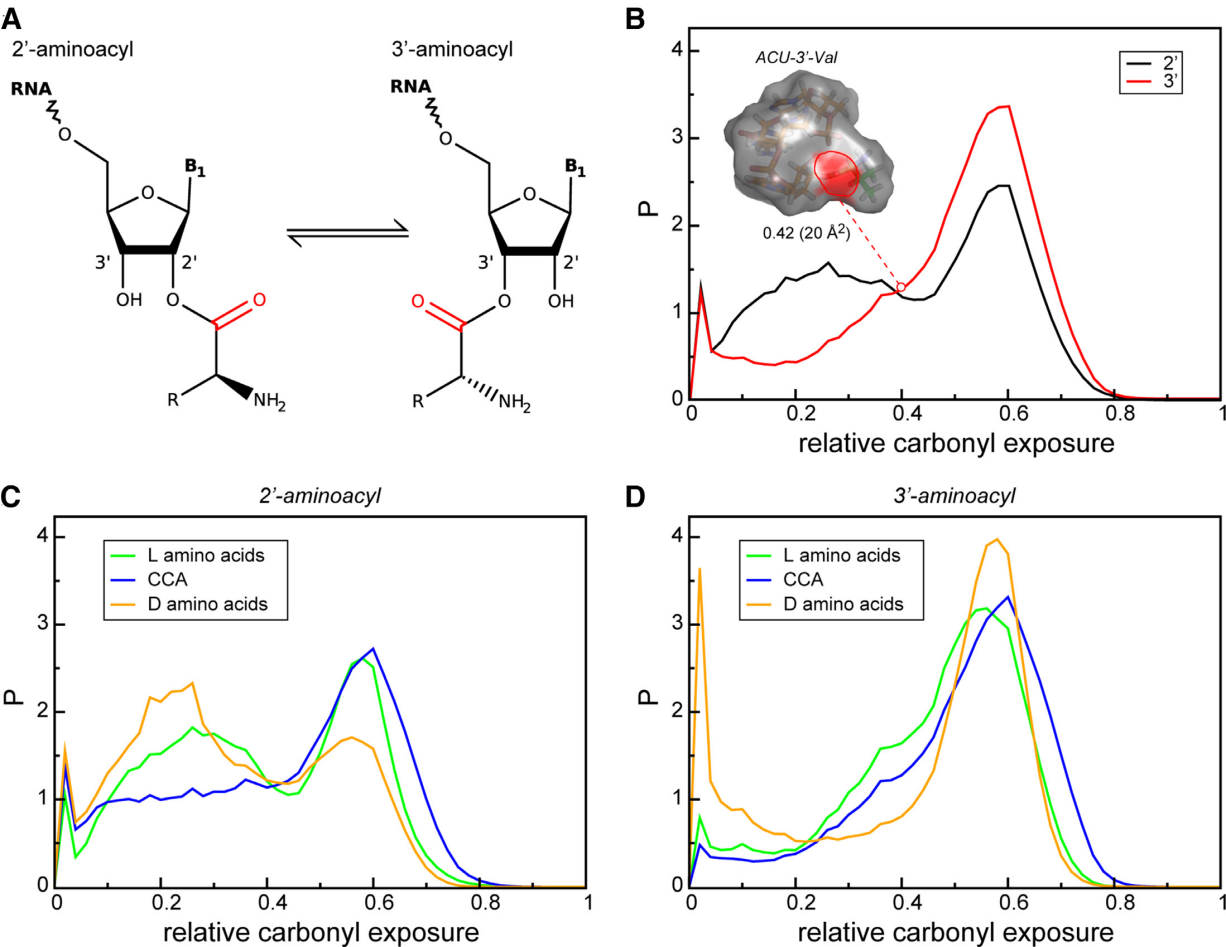
Carbonyl solvent exposure depends on the position of aminoacylation. (**A**) A schematic representation of 2′- and 3′ aminoacylated RNAs. The carbonyl group is highlighted with red. (**B**) Distribution of relative aminoacylester carbonyl solvent exposure among all simulated trajectories for 2′- (black) and 3′-aminoacylated trinucleotides (red). (C, D) Distribution of relative carbonyl solvent exposure for L-amino acids (green), CCA with different amino acids attached (blue) and D-amino acids (orange) in the case of (**C**) 2′- and (**D**) 3′-aminoacylation.

Regardless of the site of primary attachment, transacylation between the 2′- and 3′-isomers in charged tRNAs occurs readily in water via an orthoester intermediate (Figure 1A). This conversion involves a small free-energy difference (3,12): at equilibrium, the 3′-isomer is favored over the 2′-isomer by a factor of ∼3-fold, with a 2′ to 3′ conversion rate of 3–11 s^−1^ and a 3′ to 2′ conversion rate of 1–4 s^−1^ (3,12). Despite the minor energetic separation between the two isomers, most components of the translation apparatus are designed so as to stabilize and exploit either the 2′- or the 3′-aminoacylated forms of tRNA and, in this sense, go against the natural tendency toward a balanced partitioning between the two (3,13). For example, Leu- and Val-tRNA synthetases hydrolyze mischarged tRNAs exclusively from the 2′ position (10,11). On the other hand, the invariable binding of charged tRNA to an elongation factor EF-Tu (14–17), a protein responsible for transporting the tRNA to the ribosome, results in the stabilization of the 3′ isoform, which as such participates in all downstream reactions, including acceptor activity in the ribosomal A site, translocation and donor activity in the P site and release of the deacylated P-site tRNA (1–3,18–20). The exact physicochemical reasons for such a unification of charged tRNAs by EF-Tu remain, however, unclear. Specifically, does the exact site of aminoacylation affect the local conformational properties of tRNA and in this way also influence the hydrolytic stability of the aminoacylester linkage?

While all tRNAs invariably have a single-stranded CCA triplet at the end of the helical acceptor stem (positions 74–76 in the canonical tRNA numbering scheme), the hydrolytic stability of their aminoacylester linkages varies significantly as a function of the chemical nature of the attached amino acid only, as recently shown for 12 standard aminoacyl-tRNAs (21). For example, the half-life for spontaneous hydrolysis of the most stable Ile-tRNAs (τ_1/2_ = 810 min) is an order of magnitude greater than those of the least stable Pro-tRNA (τ _1/2_ = 36 min) or Gln-tRNA (τ _1/2_ = 93 min). Moreover, among all the variables tested, only the binding of the elongation factor EF-Tu significantly stabilizes the tRNA aminoacylester linkage against hydrolysis (21). Effectively, the identity of the attached amino acid modulates the stability of its ester linkage to tRNA, while this idiosyncrasy is overruled by the binding of EF-Tu. A key open question in this regard concerns the nature of the interactions between the tRNA CCA terminus and the attached amino acid. What makes some aminoacylester linkages more hydrolytically stable and some less?

Finally, it is possible that the stability of the aminoacylester linkage could have influenced the choice of CCA as the universal acceptor triplet in tRNAs. It is, therefore, important to also explore alternative choices for the acceptor triplet. This may also be critical when it comes to the question of the origin of the modern biological translation apparatus. In particular, models of primordial translation proposed by Altstein (22,23) and Sutherland & Blackburn (24) include aminoacylated RNA trinucleotides as a key element and involve RNA-templated polymerization yielding both peptide products and complementary copies of the RNA template. Providing some support for such models, templated polymerization of 2′-acylated oligoribonucleotides has recently been demonstrated (25). Importantly, the intrinsic conformational preferences of different combinations of nucleotides and amino acids in the covalent complexes, their dependence on the site of aminoacylation and their hydrolytic stability are arguably the central microscopic elements of the above proposals, but they have never been explored in detail.

Motivated by the above challenges, we have used extensive molecular dynamics (MD) simulations to study the effect of 2′- and 3′-aminoacylation on the local structure, dynamics, interactions and solvent exposure of a series of aminoacylated trinucleotides including both the canonical CCA triplet and a number of judiciously chosen alternatives. Our analysis reveals robust, systematic biases in both solvent exposure of the aminoacylester bond (the carbonyl group) in the modified trinucleotides and their conformational behavior as a function of the aminoacylation site and the chemical nature of the amino acid and the nucleotides involved.

## MATERIALS AND METHODS

### System preparation

The fully extended starting conformations of aminoacylated and non-aminoacylated trinucleotides were constructed automatically using AMBER tools 15 (*NAB*) (26) and scripts especially written for this purpose. The fully extended starting conformations were chosen in order to avoid any systematic conformational biases. In these conformations, the three bases were aligned with a slight twist to avoid stacking and, in the case of the modified trinucleotides, the attached amino acid was co-aligned with the 5′-3′ vector of the nucleotide backbone. All systems were subsequently subjected to short energy minimization *in vacuo* to allow the molecules to settle into physically reasonable configurations.

### Molecular dynamics (MD) simulations

MD simulations were performed using the GROMACS 4.5.7 package (27), Amber99SB-ILDN force-field (28) and TIP3P water model (29). Parameters for the aminoacyl bond were taken and adapted from those for the fatty acid ester in phospholipids (30). For D-amino acids, the parameters of L-amino acids were used without modification except for the corresponding geometrical changes in the initial configuration. Individual aminoacylated or non-aminoacylated trinucleotides together with ∼1000 water molecules and NaCl ions at the effective concentration of 80 mM were placed in a cubic simulation box with 3.2 nm sides. An effective pH in all systems was set to 7.

A twin-range (12/14 Å) spherical cut-off was used to truncate van der Waals interactions. Electrostatic interactions were treated using particle-mesh Ewald summation (real space cutoff 10 and 1.2 Å grid with fourth-order spline interpolation). The production MD simulations were carried out using 3D periodic boundary conditions in the isothermal−isobaric (NPT) ensemble with an isotropic pressure of 1 bar and a constant temperature of 298 K. The pressure and temperature were controlled using V-scale thermostat (31) and Berendsen barostat (32) with 0.1 and 1 ps relaxation parameters, respectively, and a compressibility of 4.5 × 10^−5^ bar^−1^ for the barostat. Trinucleotides and solvent (water and ions) were coupled separately. Bond lengths were constrained using LINCS (33).

After minimization using the steepest descent algorithm in water (100 000 steps), the systems were equilibrated for 1 ns in the NPT ensemble with position restraints (1000 kJ mol^−1^ nm^−2^) placed on each solute atom, whereby the initial 0.05 ns entailed annealing from 5 K to the reference temperature of 298 K. All production runs, each 100 ns long, were performed in the NPT ensemble, with 10 independent runs (for a total of 1 μs) for each simulated system.

### MD analysis

The relative carbonyl solvent exposure, expressed as a number between 0 and 1, was determined by normalizing the instantaneous value of the carbonyl group solvent accessible surface area (SASA) of a given conformer by 48.4 Å^2^, the maximum value observed in all of our simulations of aminoacylated trinucleotides (seen for GGU-2′-Gly). The carbonyl SASA was calculated using the *g_sas* utility from the GROMACS package (with the radius of the solvent sphere of 0.14 nm and 196 dots per sphere).

The dominant conformations of the aminoacylated trinucleotide backbone were identified by performing conformational clustering on two master ensembles obtained by joining together and subsampling either all 2′-systems (a total of 29 μs, snapshots taken every 0.1 ns) or all 3′-systems (a total of 24 μs, snapshots taken every 0.1 ns) MD trajectories (nucleotide + amino-acid backbone only). Conformational clustering on the subsampled master ensembles was performed by using the GROMACS *g_cluster* utility with a stringent backbone RMSD cut-off of 0.5 Å defining the cluster perimeter. The centers of these two dominant clusters were furthermore used to estimate the populations of related structures in each fine-grained individual MD trajectory (snapshots taken every 0.001 ns) by calculating the fraction of conformations with the backbone RMSD from the cluster-center structure ≤1.5 Å (*g_rms*, GROMACS). Note that in this analysis, each individual conformer was assigned to either one of the two top cluster centers or the rest (Figure 4A-B; see Supplementary Master Supplementary Table S1 for the specific fractions of these conformational states in each individual system). The same analysis was performed for unmodified trinucleotides, but using only nucleotide backbone atoms of the reference structures. Finally, the same procedure was applied to identify the dominant conformations observed in the simulations of individual CCA trinucleotides in the MD ensembles of the CCA terminus of a full tRNA. For this purpose, three independent MD runs (0.333 μs each) of the yeast tRNA^phe^ from the study by Zhang *et al.* (34) were analyzed. Note that the MD protocol in that study is similar to the one applied here and also employed Amber99 force field.

The joint 2′- and 3′-master ensembles were further analyzed by performing the principal component analysis (PCA) on the *x*, *y* and *z* coordinates of backbone atoms by using the GROMACS *g-covar* utility. The individual MD trajectories of AA3Ns were then projected onto the generalized coordinates along the first and the second eigenvectors (*g_anaeig*, GROMACS).

Base-base stacking and amino-acid sidechain-base contacts in aminoacylated and non-aminoacylated trinucleotides were defined by using a distance cutoff between the corresponding centers-of-geometry (COG) of <5 and <8 Å (35), respectively. All calculations were performed using the *g_dist* utility from the GROMACS package.

### Statistical analysis

Multiple regression analysis of the dependence between the relative carbonyl exposure or the relative preferences of collapsed and extended states and a set of key intramolecular interactions used as predictors (see Supplementary Table S1, columns 3–8) was performed by using *regstats* function in MATLAB (R2009). The *P*-values for multiple regression analysis were obtained using *F*-test, performed under the assumption that the model contains a constant term. To further assess the contribution of each predictor, a Proportional Reduction in Error (PRE) test was performed, as described elsewhere (36). The PRE value, reported for a given predictor, captures the relative amount by which the residual sum of squares in a linear model is reduced if one adds a given predictor to a model involving all the other predictors, i.e. the higher the PRE value, the more significant the contribution of a given predictor. The *P*-values for simple linear regression were obtained using *t*-test.

## RESULTS

### Simulated systems

The total number of combinations of canonical amino acids and RNA trinucleotides exceeds 1200. In order to approach this vast combinatorial space systematically, we have selected three classes of systems for simulation (Table 1). These include: (i) the same amino acid attached to different trinucleotides (the valine set), (ii) the same trinucleotide attached to different amino acids (the CCA set) and (iii) a representative subset of amino acids attached to their select cognate codons (the cognate set). Valine was chosen because its sidechain is able to form only transient van-der-Waals contacts with the nucleotides, allowing one to study the role of steric interactions in defining the conformational preferences of aminoacylated trinucleotides. Moreover, valine is considered to be one of the earliest amino acids used in primordial biological systems (37). The valine set includes the valine GUU codon, its respective wobble anticodon CAU and their scrambled variants UGU and ACU in all combinations of 2′- and 3′-aminoacylation and d- and l-enantiomeric states (Table 1). The aminoacylated CCAs were chosen because of their relevance for tRNA biology and the possibility to validate our computational models by comparing against the experimentally determined stabilities of aminoacylated tRNAs (21). The CCA set includes different combinations of 2′ and 3′ attachment of CCA to glutamate, glycine and twelve other amino acids for which tRNA hydrolysis half-lives are available (21) (Table 1). Finally, we have also simulated a set of amino acids with different physicochemical properties (arginine, aspartate, glutamate, glycine, proline, threonine and valine) attached to 2′ positions of their corresponding codons with uracil or adenine in the third position to be consistent with the valine and the CCA sets. Additionally, glutamate was also simulated as a D-enantiomer and in 3′ attachment with its cognate GAA codon. Most trinucleotides were also simulated in a free form as controls (Table 1). For each system, the total simulated time was 1 μs (10 × 100 ns independent MD simulations with snapshots taken every 1 ps for each). This protocol allowed us to sample well the available conformational space for both positions of amino-acid attachment and both amino-acid enantiomers (Supplementary Figure S1). Altogether, we have collected 53 μs of MD statistics for aminoacylated trinucleotides and 6 μs for free trinucleotides. Finally, we have compared our results against the 1 μs of MD sampling for tRNA^Phe^ from a study by Mathews and coworkers (34).

**Table 1.** Simulated systems

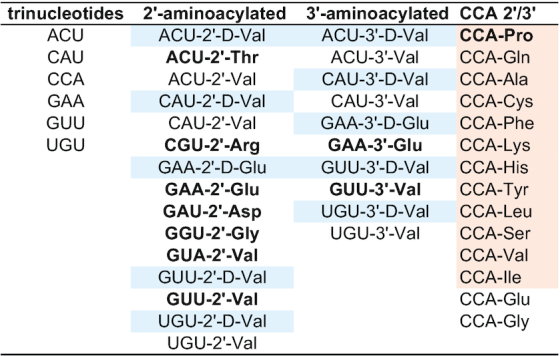

*Cognate pairs of amino acids and their codons are shown with bold characters, constructs containing D-amino acids are highlighted in pale blue, while CCA-modified trinucleotides corresponding to the experimental study of tRNA hydrolytic stabilities by Peacock *et al.* (21) are highlighted in pale orange.

### Solvent exposure of aminoacylester bond as a function of stereochemistry

Analysis of MD trajectories of simulated 2′- and 3′-modified trinucleotides shows that the aminoacylester bond is significantly more solvent exposed in the case of 3′-aminoacylated systems. Specifically, the joint distribution of the relative solvent exposure of the aminoacylester carbonyl group, as determined over all simulations, is significantly shifted towards higher values in the case of 3′ as compared to 2′ attachment (Figure 1B). While the 3′ systems exhibit a single strong peak in the relative solvent exposure of the carbonyl group at 0.6, the population of 2′ systems is split between two pronounced peaks, centered at ∼0.2 and 0.6 (Figure 1B). This, in turn, significantly lowers the average exposure in the case of 2′ systems. In addition to the attachment site, the composition of trinucleotides and amino-acid chirality also govern the solvent exposure of the aminoacylester carbonyl group. Most importantly, the carbonyl group tends to be more exposed in CCA trinucleotides, both 2′ and 3′, as compared to other modeled aminoacylated systems (Figure 1C and D). Specifically, the peak in the relative solvent exposure at ∼0.2 in 2′-modified systems is much lower and flatter for CCA trinucleotides regardless of the chirality of the attached amino acid (Figure 1C). Equally importantly, both 2′ and 3′ CCA systems exhibit a higher population of states with a particularly high solvent exposure as compared to other trinucleotides (Figure 1C and D). At the same time, for both types of aminoacylation, the D-amino acids exhibit a higher population of conformational states with a low carbonyl solvent exposure as compared to L-amino acids (Figure 1C and D).

**Figure 2.**
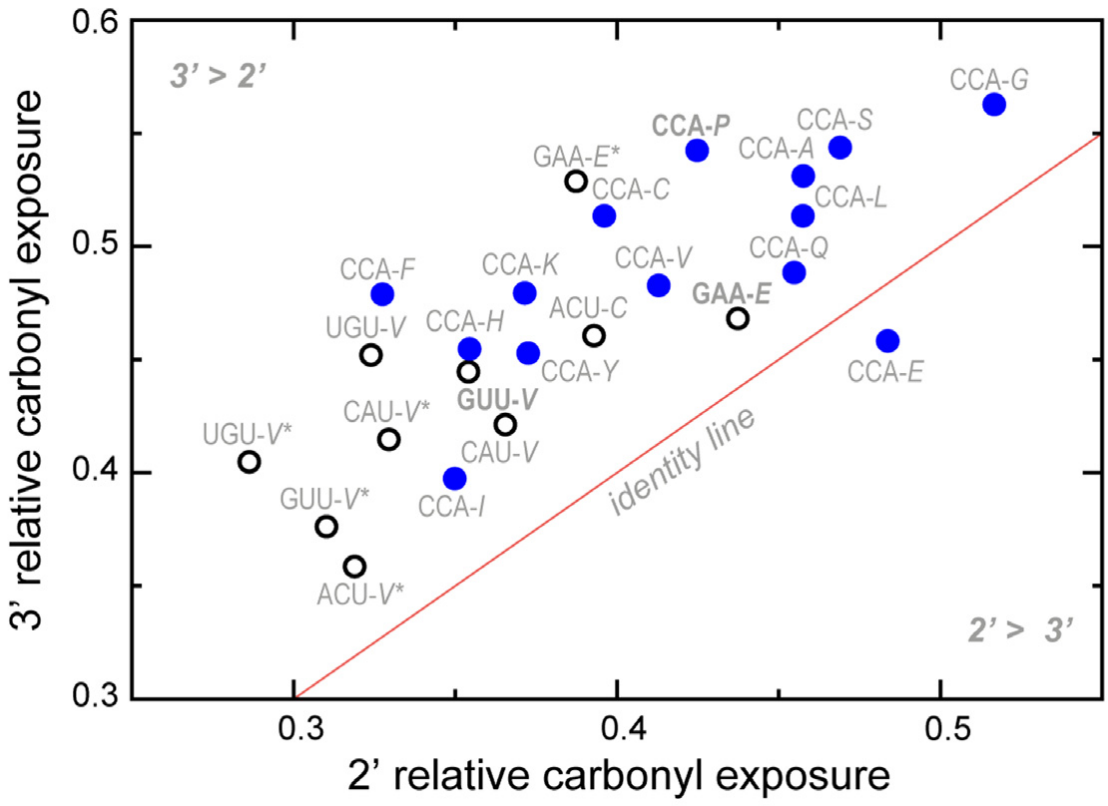
Carbonyl solvent exposure in different systems. Average relative carbonyl exposure of 2′- and 3′-isomers in the case of CCA (blue) and non-CCA (open black) trinucleotides with different amino acids attached. D-amino acids are labeled with a star. Aminoacylated trinucleotides involving cognate pairs of amino acids and their codons are labeled in bold.

In Figure 2, we compare the calculated levels of aminoacylester carbonyl solvent exposure in individual 2′- and 3′-aminoacylated trinucleotides. As is evident, carbonyl solvent exposure in the great majority of 3′-aminocylated isomers exceeds that in their 2′ counterparts, with CCA-Glu being the only exception. Furthermore, we observe a pronounced grouping of simulated systems with respect to their composition. Thus, the majority of aminoacylated CCA systems, regardless of the site of attachment, display higher solvent exposure of the aminoacylester bond as compared to other simulated systems (Figure 2).

### Intramolecular interactions tune carbonyl solvent exposure

**Figure 3.**
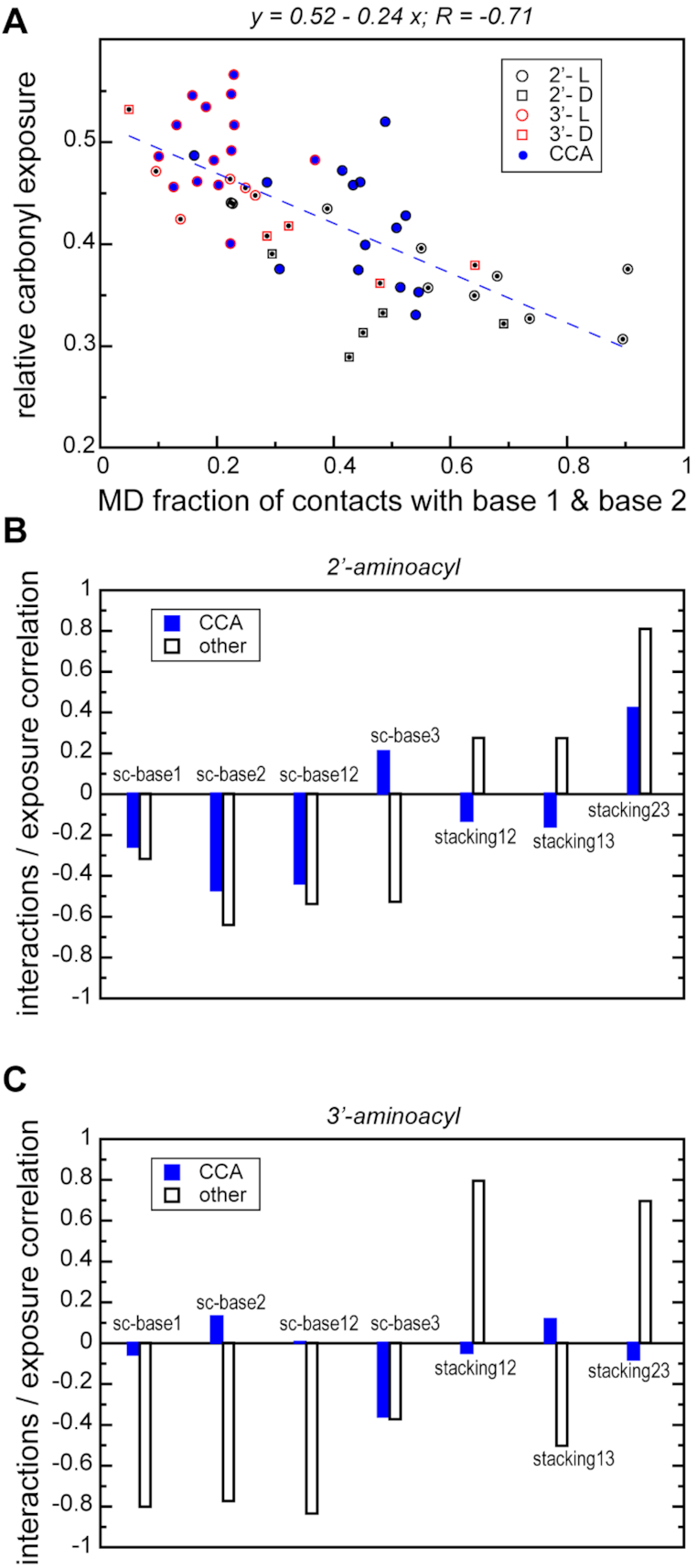
Interactions tune carbonyl solvent exposure. (**A**) An exemplary linear regression between the average relative carbonyl solvent exposure and sidechain interactions with the first and the second bases estimated as MD fractions of distances < 8 Å between sidechain and base centers-of-geometry (COGs). The results for L- and D-aminoacylated trinucleotides are depicted with circles and rectangles, respectively, while for 2′- and 3′-modified trinucleotides they are shown in black and red, respectively. (**B**, **C**) Pearson correlation coefficients between the sidechain interaction strengths with different bases or the stacking between bases and the average relative carbonyl solvent exposure in the case of 2′-modified (B) and 3′-modified trinucleotides. The open and blue bars correspond to non-CCA and CCA trinucleotides, respectively.

We next analyzed the connection between different intramolecular interactions in aminoacylated trinucleotides and the solvent exposure of the aminoacylester carbonyl group. Specifically, we have analyzed the dependence of the carbonyl exposure levels on the establishment of either close contacts between the sidechain and the bases or stacking interactions between different pairs of bases (see Master Supplementary Table S1 for detailed values in each system). Regardless of the amino-acid attachment site, the frequency of conformations in which the sidechain simultaneously interacts with the first and the second base is inversely correlated with carbonyl exposure (*R* = –0.71, *P*-value < 0.00001; Figure 3A). However, there appears to be a major difference between CCA and non-CCA trinucleotides in this regard. Specifically, carbonyl exposure in aminoacylated CCA trinucleotides, especially in the 3′ case, is significantly less correlated with intra-molecular interactions such as sidechain-base contacts and base-base stacking as compared to other modeled systems (Figure 3B and C). For example, the level of carbonyl exposure in 3′-aminoacylated non-CCA trinucleotides exhibits a strong inverse correlation with the level of interaction of the sidechain with the first and the second bases (*R* = –0.84, *P*-value = 0.00235; Figure 3C). At the same time, carbonyl exposure in 3′-aminoacylated CCAs is completely unrelated to different intra-molecular interactions (Figure 3C). Similarly, a significant correlation with carbonyl exposure in 2′-aminoacylated non-CCAs is observed for stacking interactions between the second and the third bases (*R* = 0.81, *P*-value = 0.00025; Figure 3B). Again, aminoacylated CCAs display no significant correlation between stacking interactions and carbonyl exposure.

The results of the above simple regression analysis are corroborated by a multiple regression analysis with the amino-acid interactions with bases 1, 2 and 3 together with the stacking between bases 1 and 2, 2 and 3 and 1 and 3 taken as predictors (Supplementary Table S3). Most importantly, a linear model combining all key intramolecular interactions provides the best fit for 2′-aminoacylated non-CCA trinucleotides (*R*^2^ = 0.81, *P*-value = 0.0131), whereby the strongest contribution comes from stacking interactions between base 2 and base 3, confirming the findings of the simple regression analysis. Moreover, the carbonyl exposure in CCA trinucleotides, both 2′ and 3′, exhibits a weak dependence on the intramolecular interactions (Supplementary Table S3), as in the simple regression analysis. Altogether, these results underscore the trend that the adoption of conformations, in which the amino acid *folds back* towards the 5′ end of the trinucleotide and the sidechain interacts directly with the first and the second base, helps reduce solvent exposure. In contrast, stacking between neighboring bases negatively affects such folding and maintains a high level of carbonyl group exposure to water. Surprisingly, modified CCA trinucleotides display a significantly lower level of correlation between carbonyl exposure and intra-molecular interactions, especially in the case of 3′-attachment.

### Structural correlates of carbonyl solvent exposure

**Figure 4.**
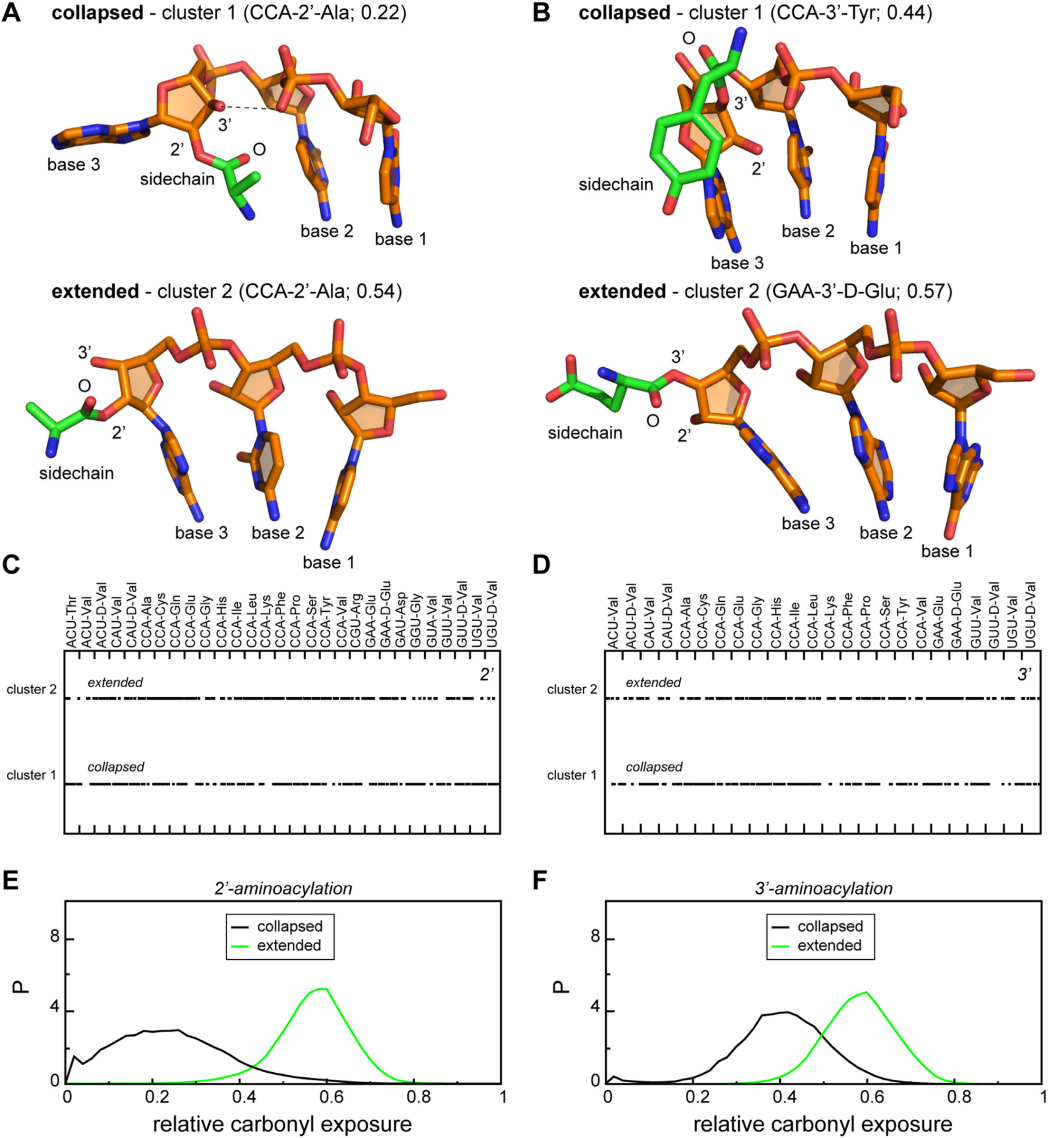
Structural correlates of carbonyl solvent exposure. (A, B) The top 2 most populated structural clusters of aminoacylated trinucleotide backbone conformations in the case of 2′ (**A**) and 3′ (**B**) attachment. Cluster centers are given in a stick representation. (C, D) Distribution of the dominant conformations along MD trajectories of (**C**) 2′- and (**D**) 3′-aminoacylated trinucleotides. (**E-F**) Relative carbonyl solvent exposure in different conformational states of (**E**) 2′- and (**F**) 3′-aminoacylated trinucleotides.

The wide distributions of relative carbonyl exposure shown in Figure 1 indicate a high degree of structural diversity in simulated systems. In order to provide a structural background for different carbonyl exposure levels, we have performed RMSD-based clustering of backbone conformations for joint MD trajectories combining separately all simulated 2′ and 3′ systems using a stringent cluster cutoff (0.5 Å; see Materials and Methods for details). In Figure 4A and B, we show the central members of the two most populated conformational clusters obtained in this way for 2′- and 3′-modified trinucleotides, respectively. In recognition of their structural features, we label these conformations *collapsed* and *extended*.

**Figure 5.**
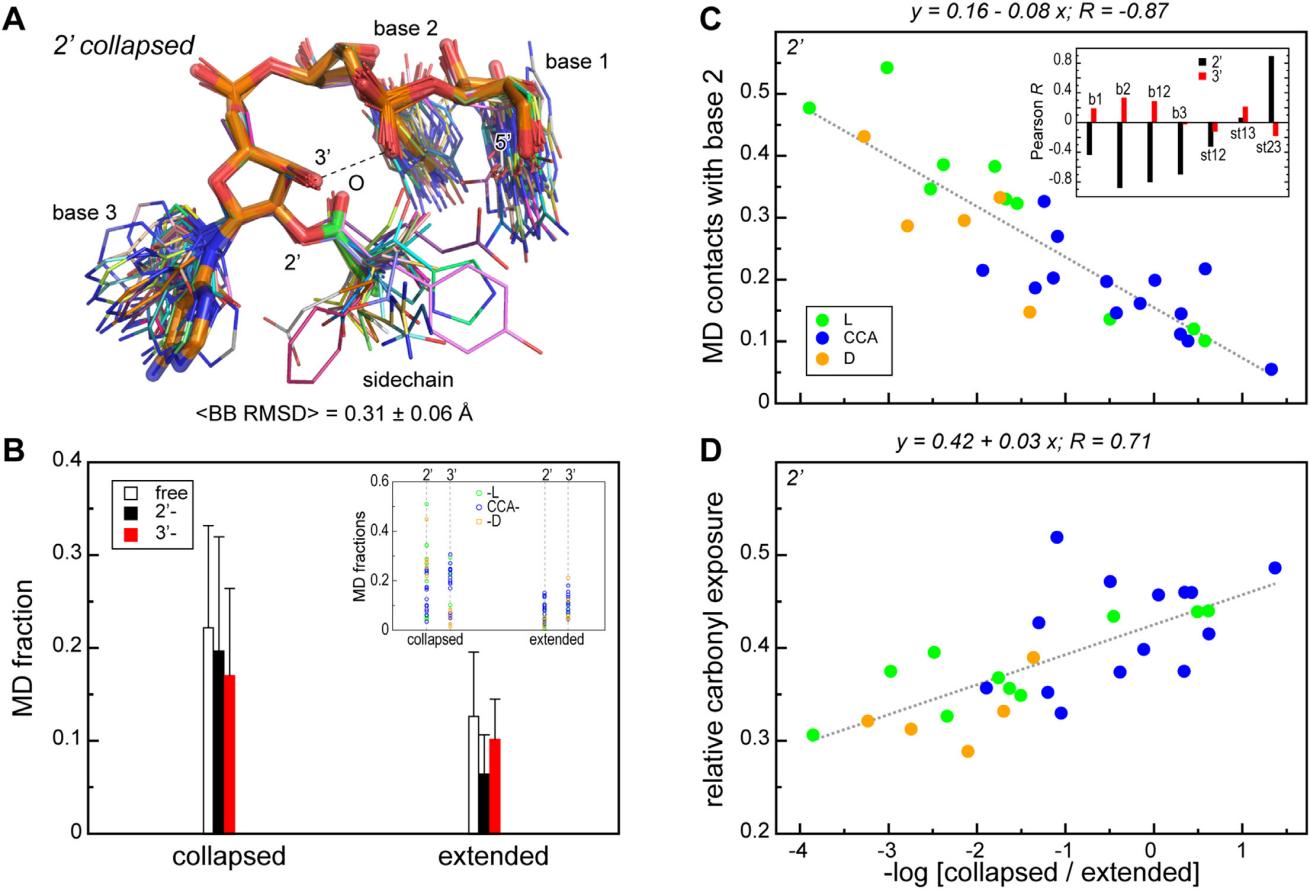
Dominant conformational states of aminoacylated trinucleotides. (**A**) Superposition of the representative 2′-aminoacylated *collapsed* states for all simulated systems. The reference conformation for the fitting (CCA-2′-Ala *collapsed*) is shown with thick transparent sticks. (**B**) Average MD fractions of the dominant conformational states for free (open bars), 2′- (black bars) and 3′-aminoacylated (red bars) trinucleotides. The thin lines depict standard deviations. *Inset*: MD fractions of the dominant conformational states for each individual system. L-amino acids, CCA with different amino acids attached, and D-amino acids are shown with green, blue and orange circles, respectively. (**C**) An exemplary linear regression between the MD fraction of close contacts of sidechain with the second bases in 2′-aminoacylated nucleotides and the conformational preference for the *collapsed* over the *extended* state. *Inset*: Pearson correlation coefficients *R* between the conformational preference and different intra-molecular interactions shown with black and red bars for 2′- and 3′-modified systems, respectively. (**D**) A linear regression between the conformational preference in 2′-modified trinucleotides and the respective average relative carbonyl solvent exposure.

The globally most populated 2′ conformation (*collapsed*) exhibits a folded-back geometry whereby the first and the second base form a stacking pair, the third base is flipped out and the sidechain is oriented towards the 5′-hydroxyl of the first nucleotide forming direct contacts with the second and, to a lesser extent, the first base (Figure 4A). Additionally, in this conformation, the 3′-hydroxyl of the third nucleotide is in a perfect arrangement for H-bonding with the phosphate oxygen of the second nucleotide, providing stabilization. Importantly, the *collapsed* state is present in every individual MD ensemble of 2′-modified trinucleotides (Figure 4C) and has an invariant backbone geometry. The latter can be illustrated by superimposing individual conformations from different 2′-modified systems that are closest in an RMSD sense to the reference *collapsed* structure obtained by clustering the combined master trajectory (the collapsed conformation of CCA-2′-Ala). Remarkably, the average backbone RMSD for an all-to-all comparison in this case is only 0.31 Å (Figure 5A). At the same, the relative positions of the sidechain and the bases display some flexibility, depending on the type of the amino acid and the nucleotides. Concerning occupancy, for some 2′-modified trinucleotides, >40% of all simulated conformations belong to the *collapsed* conformation, with an average occupancy over all individual 2′ systems being 20 ± 13% (Supplementary Table S1, Figure 5B). Importantly, the *collapsed* conformation is a member of the most populated cluster for 19 out of 29 individual 2′-modified systems (Supplementary Table S2).

The second most populated conformational cluster of the joint 2′ trajectory corresponds to an *extended* conformation with an average fractional population of 6.6 ± 4% per individual 2′ system (Figure 5B, Supplementary Table S1). Moreover, 17 out of 29 individual 2′ systems have this conformation among their most 5 populated clusters (Supplementary Table S2). In the *extended* conformation, the three bases stack and the sidechain extends into the solution, displaying no significant interactions with the nucleotides (Figure 4A). Importantly, due to the geometry of folding, the identified conformations differ significantly in the level of carbonyl solvent exposure (Figure 4E). Specifically, the *extended* conformation exhibits the highest exposure, which provides little or no protection to the aminoacylester bond: the joint distribution of aminoacylester carbonyl solvent exposure for all 2′ systems in the *extended* conformation is significantly shifted towards high values, peaking at around 0.6 in a rather system-independent manner (Figure 4E). At the same time, the dominant *collapsed* state displays a wide distribution of exposure values without a clearly defined maximum (Figure 4E), albeit still shifted towards low levels. In other words, in the dominant *collapsed* state, a direct interplay between the sidechain chemical structure and nucleotide composition, which defines the intramolecular interaction strength, also shapes the solvent exposure of the aminoacylester bond.

Analysis of other structural clusters for the joint 2′ trajectory shows that their occupancy drops significantly as a function of cluster rank (Supplementary Figure S2B). Thus, clusters with ranks from 3 to 5 have populations of a few percent and adopt structures with different possibilities for carbonyl exposure. For example, the clusters 4 and 5 contain states that can be characterized as intermediates between the *collapsed* and the *extended* states, while cluster 3 represents an alternative collapsed, folded-back conformation—*collapsedinverted* (Supplementary Figure S2A). Here, the sidechain approaches the first two bases from the other side in relation to the 5′-hydroxyl as compared to the dominant *collapsed* state, which makes the establishment of direct contacts with the corresponding bases less geometrically probable, but provides a prominently low level of carbonyl exposure (0.15 ± 0.05 on average).

Remarkably, in the case of 3′ aminoacylated trinucleotides, we identify the same two dominant conformational states with identical nucleotide backbone scaffolds as for the 2′ systems: 1. *collapsed* and 2. *extended* (Figure 4B). Most importantly, the *collapsed* conformation still dominates with an average fractional population of 17 ± 9% per individual 3′ system (Figure 5B, Supplementary Tables S1 and S2) and 15 out of 24 individual 3′ systems having this conformation in their most populated cluster (Supplementary Table S2). The *extended* state becomes more populated for 3′ aminoacylation (Figure 5B, Supplementary Table S1) with an average fractional population of 10 ± 4% per individual 3′ system (Figure 5B, Supplementary Table S1) and 19 out of 24 individual 3′ systems having this conformation among their most 5 populated clusters (Supplementary Table S2). Here, it is important to note that the geometry of amino-acid attachment defines a different orientation of the sidechain with respect to nucleotides in the dominant *collapsed* state (Figure 4B). Here, in stark contrast to 2′ systems, the aminoacylester carbonyl exhibits preferential orientation toward the solvent and maintains high exposure in most cases, with the maximum of the joint distribution at ∼0.40 (Figure 4F). Moreover, in this case, the free 2′-hydroxyl is not able to form a hydrogen bond with phosphate oxygens of the second base, reducing the MD population of this state as compared to the 2′ isomer (Figures 4D and 5B). Also, the geometry of the amino-acid attachment makes a direct contact between the first two bases and the sidechain less probable (Figure 4B). Interestingly, extended states of 3′-aminoacylated trinucleotides exhibit a similar distribution of carbonyl exposure values as their 2′ counterparts (Figure 4F). Other highly occupied structural clusters for the joint 3′ trajectory display similar trends as in the 2′ case (Supplementary Figure S2B). Here, clusters 3 and 4 also resemble intermediate states between the *collapsed* and *extended* states with a high level of carbonyl exposure, while cluster 5 corresponds to the *collapsedinverted* state identical to that in the 2′ case (Supplementary Figure S2A). Of the top 5 clusters in the case of 3′-attachment, only the latter cluster exhibits a low level of exposure of the carbonyl (0.14 ± 0.1 on average).

### Intrinsic conformational statistics of unmodified trinucleotides

Does the attached amino acid define the dominant conformational states in modified trinucleotides or is the preference for a given conformation an intrinsic property of a given nucleotide composition? We have analyzed the populations of the *collapsed* and *extended* states in MD simulations of unmodified trinucleotides using a backbone RMSD distance from the nucleotide backbone scaffolds of the reference conformations (see Methods for details). Interestingly, we observe similar statistics of the *collapsed* and *extended* states, ordered here with respect to their average populations (Figure 5B, see Supplementary Table S1 for detailed fractions in each system), in the two cases. Thus, aminoacylation does not induce folding of trinucleotides into the above dominant conformations, but rather affects their populations. In particular, amino-acid attachment to the 2′-hydroxyl does not change the average fraction of the *collapsed* state (Figure 5B), while it does increase the variability among different systems. This is especially prominent for the non-CCA L-amino acid constructs (Figure 5B, inset). At the same time, amino-acid attachment to the 3′-hydroxyl systematically depletes the *collapsed* state due to the lack of the stabilizing H-bond between the 3′-hydroxyl and phosphate oxygens of the second base (see above). The *extended* state is significantly less populated in 2′-aminocylated systems as compared to free trinucleotides due to a perturbation of base stacking interactions through contact formation with the sidechain. This effect is less pronounced for 3′-attachment, since in that case the geometry of the attachment is less optimal for specific sidechain-base interactions (see above). Finally, different unmodified trinucleotides exhibit different intrinsic preferences: CCA and especially GAA prefer extended, while other trinucleotides prefer collapsed conformations (Supplementary Figure S3A). Furthermore, these preferences are directly related to stacking interactions, which are composition dependent. Thus, stacking between base 2 and base 3 strongly correlates with the preferences (*R* = 0.84, *P*-value = 0.03635, Supplementary Figure S3B) suggesting that the stronger the stacking between these bases, the more the trinucleotide prefers the extended state. An opposite, but less pronounced trend is observed for stacking between bases 1 and 3 (*R* = –0.70, *P*-value = 0.1215, Supplementary Figure S3B). Altogether, multiple regression analysis shows that stacking interactions are good predictors of the conformational preference in trinucleotides (*R*^2^ = 0.97; *P*-value = 0.049; Supplementary Figure S3B, inset).

### Relative preferences of collapsed and extended states

Although collapsed conformations dominate over extended ones in most cases, the conformational preferences of individual aminoacylated trinucleotides display some specificity when it comes to both trinucleotide composition and the chemical nature of the amino acid involved. We express the preference for collapsed over extended conformations in individual systems as a negative logarithm of the ratio of the occupancies of the two states, a proxy of their free energy difference (Figure 5C and D). In the case of 2′-aminoacylation, the thus derived preferences are directly related to the frequency of interaction between the sidechain and the bases. This effect is strongest for the second base, whereby a high frequency of contacts between the sidechain and the second base is indicative of collapsed conformations and *vice versa* (*R* = –0.87, *P*-value < 0.00001; Figure 5C). At the same time, 2′-aminoacylated trinucleotides prefer being *collapsed* over *extended* if the stacking between base 2 and base 3 is weak and *vice versa* (Figure 5C, *inset*). Interestingly, due to the different geometry of amino-acid attachment, no similar trends are observed in the case of 3′ systems (Figure 5C, inset). In-line with the simple regression analysis, multiple regression analysis (Supplementary Table S3) clearly shows that intramolecular interactions are a much stronger determinant of the relative conformational preferences in 2′-aminoacylated trinucleotides (*R^2^* = 0.68, *P*-value = 0.0002) than in their 3′ counterparts (*R*^2^ = 0.12, *P*-value = 0.868). In the former case, the best fit is found for 2′-aminoacylated non-CCA trinucleotides (*R*^2^ = 0.81, *P*-value = 0.0136), whereby the strongest contribution comes from stacking interactions between base 1 and base 2, with additional strong contributions of stacking interactions between base 1 and base 3 and sidechain interactions with the middle base (Supplementary Table S3).

Importantly, in the case of 2′-aminoacylation, the preference for being *collapsed* over *extended* is directly related to the degree of solvent exposure of the carbonyl group (*R* = 0.71, *P*-value = 0.00002; Figure 5D). Thus, CCAs 2′-modified by L-amino acids exhibit a higher frequency of extended conformations and a higher carbonyl exposure level, trinucleotides 2′-modified by D-amino acids show the opposite trend and no such biases are observed for L-amino acids in combination with non-CCAs. Again, no strong relationship between the preference for being collapsed over extended and the carbonyl exposure is seen for 3′ systems (*R* = 0.008, *P*-value = 0.97, not shown).

### Collapsed and extended conformations of CCA in a full-length tRNA context

**Figure 6.**
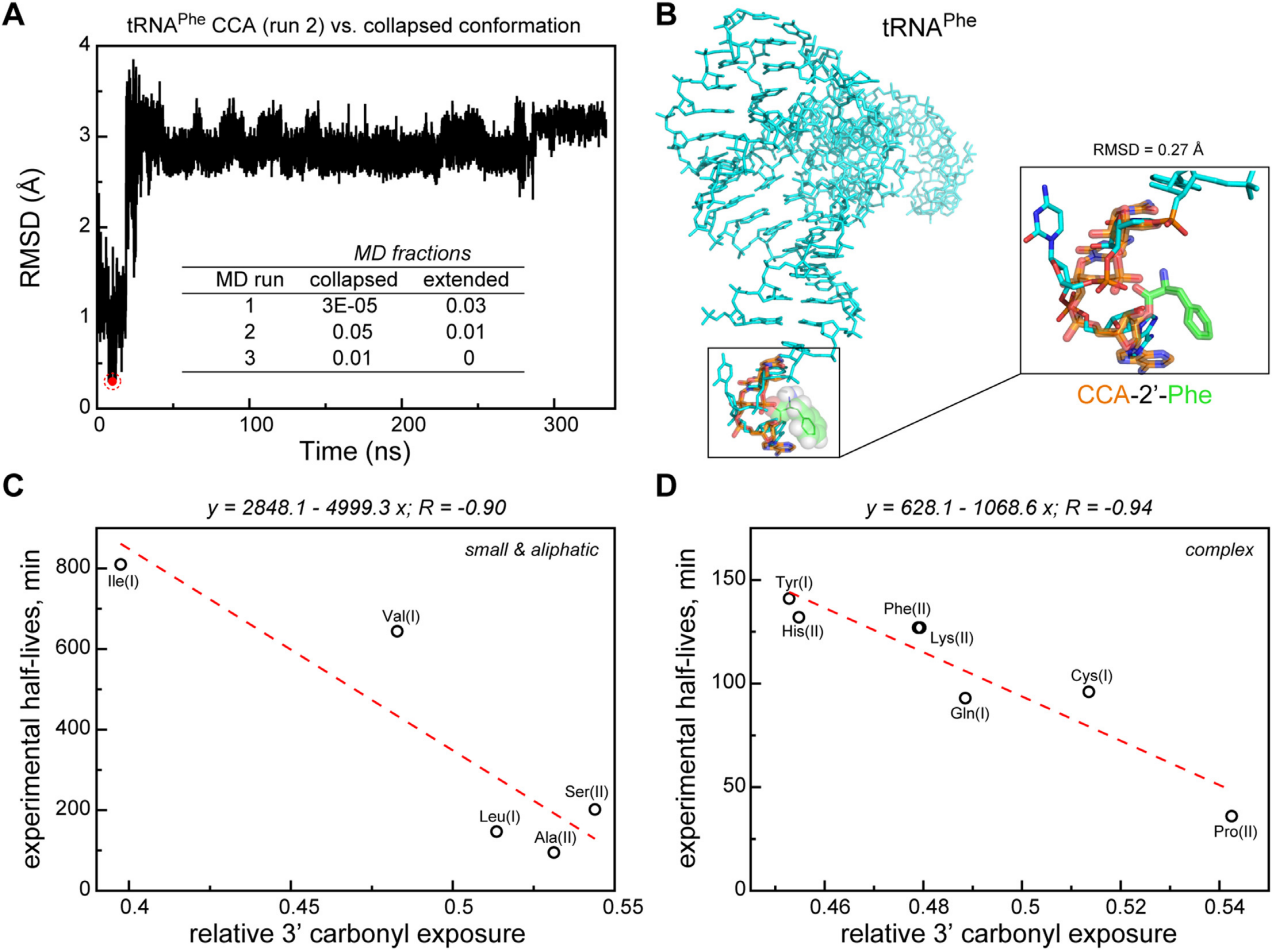
CCA conformational dynamics and carbonyl solvent exposure in a full-length tRNA context. (**A**) MD time-series of backbone root-mean-squared deviation (RMSD) for the CCA terminus of the yeast tRNA^Phe^ from the dominant *collapsed* conformation in the joint MD trajectories of aminoacylated trinucleotides. A position of CCA terminus MD-conformation closest to the collapsed state (see also panel B) is shown with a red circle. *Inset:* MD fractions of the collapsed and the extended state of the CCA terminus in different independent MD runs of the yeast tRNA^Phe^. The tRNA^Phe^ MD trajectories were taken form Zhang *et al.* (34). (**B**) Superposition of the collapsed conformation for CCA-2′-Phe and the CCA terminus of the yeast tRNA^Phe^. tRNA^Phe^ and CCA-2′-Phe are shown in stick representation. tRNA^Phe^ is shown in cyan. (C, D) Linear regressions between the average relative carbonyl solvent exposure and the experimental tRNA hydrolysis half-times (21) for small and aliphatic (**C**) and large, polar, aromatic and charged (**D**) amino-acid residues. For each amino-acid label, the corresponding aminoacyl-tRNA synthetase class is given in the parentheses.

To what extent do isolated trinucleotides, especially CCA, capture the conformational behavior of the terminal triplet in a full tRNA? In order to address this question, we have analyzed three independent MD simulations of uncharged tRNA^Phe^ (34) and probed them for the presence of the dominant collapsed and extended conformations as detected in the simulations of isolated trinucleotides (see Materials and Methods for details). As shown in Figure 6A, the CCA terminus of tRNA^Phe^ can be found in the *collapsed* and *extended* conformations at a relative frequency of 5% and 3%, respectively. This suggests that these conformational states are an intrinsic feature of CCA even in the full tRNA context. On the other hand, the exact populations of such states are strongly modulated by CCA interactions with other parts of the tRNA. Namely, uncharged CCA in MD simulations of full tRNA displays a tendency to bind directly to the tRNA body and resides in such an arrangement for most of the simulated time. We have also attempted to fit the *collapsed* conformation of CCA-2′-Phe to the respective conformation of the uncharged tRNA^Phe^ CCA terminus. Note that although charging of tRNA^Phe^ is catalyzed by class II aminoacyl-synthetase, the enzyme exceptionally attaches the amino acid at the 2′ position. We find that collapsed CCA-2′-Phe, as obtained from our simulations of the individual aminoacylated trinucleotide, can be fitted without any steric clashes and overlays perfectly with the corresponding tRNA moiety with an RMSD for the RNA backbone of 0.3 Å only (Figure 6B). Thus, dominant conformational states seen in our simulations of isolated trinucleotides are fully compatible with being realized in a full tRNA context.

### Solvent exposure in correspondence to hydrolytic stability of aminoacylated tRNAs

Hydrolytic stability is a central functionally relevant feature of aminoacylated RNAs. Since activating the carbonyl group by protonation or hydroxide nucleophilic attack (depending on the pH) is a critical step in ester hydrolysis, the rate of this reaction is related in part to solvent exposure of the carbonyl atoms. To what extent does the relative solvent exposure of the carbonyl group, as analyzed in the present study, capture the group's hydrolytic stability? In order to address this question, we have compared the relative carbonyl exposure of simulated modified CCA trinucleotides against the experimentally measured hydrolysis half-times of the corresponding aminoacylated tRNAs (21) (Supplementary Figure S4A and S4B). Interestingly, the relative carbonyl exposure values of the twelve 3′-aminoacylated CCAs for which there exists experimental tRNA data display a substantially stronger correlation with the experimental life-times (*R* = –0.62, *P*-value = 0.03151) than their 2′-counterparts (*R* = –0.25, *P*-value = 0.433). This is consistent with the fact that tRNAs in the experiment were all 3′-aminoacylated using a specific dFx ribozyme. Moreover, while transacylation between 2′ and 3′ sites occurs readily in solution, the equilibrium is shifted toward 3′ isomers by a factor of ∼3-fold (3,12). Overall, the hydrolytic-life times, as measured experimentally, and the relative carbonyl exposure values, as determined in our simulations, do not display any noticeable trends with respect to the class of the corresponding aminoacyl tRNA-synthetase (Supplementary Figure S4A and S4B). On the other hand, by performing a multiple regression analysis, we could detect a statistically strong connection between carbonyl exposure as well as conformational preferences and various interaction parameters only in the case of 2′ Class I systems (Supplementary Table S4). Finally, it is likely that the β-branched nature of Ile and Val contributes significantly to the fact that their respective aminoacyl-tRNAs exhibit the slowest experimental hydrolysis times, as suggested before (21). In fact, a simple regression analysis of the dependence between an amino acid being β-branched or not and the experimental tRNA hydrolysis times for the 12 studied systems yields a high Pearson *R* of 0.97 and a *P*-value < 10^−6^. However, it should be noted that such a high correlation is effectively caused by the two extreme values corresponding to Ile and Val and provides no explanatory mechanism for the differences in the tRNA hydrolysis times in the case of the remaining 10 non-β-branched amino acids.

On the other hand, the solvent exposure of the aminoacylester carbonyl group, which does report in part on the branching, but also other physicochemical characteristics of the amino acids in question, may be able to differentiate in a more nuanced manner between different systems. In particular, the chemical nature of the amino-acid sidechain (small and aliphatic or more complex such as aromatic, charged and polar) does seem to contribute to rationalizing the dependences between the carbonyl exposure as seen in the simulations and the experimentally obtained tRNA hydrolysis rates. Thus, the two values correlate with each other with Pearson *R*s of -0.90 (*P*-value = 0.03739) and –0.94 (*P*-value = 0.00164) for small and aliphatic amino acids, and for large polar, aromatic and charged ones, respectively (Figure 6C and D). However, the obtained regression slopes and offsets are different for these two subsets, suggesting that just conformational effects may have a different impact on the hydrolytic stability of tRNAs modified by amino acids of given type, while an additional contribution may come from the precise electronic structure of the sidechain involved, an effect which is out of the scope of the present study.

Overall, our analysis shows that carbonyl exposure can provide a realistic estimate of the relative hydrolytic stability of aminoacylated RNAs. Second, it suggests that the different hydrolytic stabilities of tRNAs may be to a large extent due to local effects, i.e. a direct interaction between the attached amino acid and the CCA part of the tRNA acceptor stem.

## DISCUSSION

Although tRNA aminoacylation is a central reaction in the process of translation, the structural and dynamic consequences of amino-acid attachment to 2′- or 3′-hydroxyls of the tRNA A76 ribose moiety have so far not been studied in atomistic detail. In the present study, we show that the site of aminoacylation directly affects the solvent exposure of the aminoacylester bond and could, thus, substantially affect its hydrolytic stability. Moreover, the aminoacylation site directly shapes the conformational dynamics of the modified trinucleotides. The obtained results deepen our understanding of the mechanistic aspects of aminoacyl-tRNA transformations in the course of translation and provide hints as to which factors could have shaped the early-stage evolution of the translation apparatus. Here, we first critically examine the methodological aspects of our study, followed by a general discussion of its principal findings.

Classical MD simulations in conjunction with semi-empirical, atomistic force fields and advanced sampling algorithms have over the past decades become a powerful, mature tool for studying the inner workings of biological systems at the atomistic level. Here, we have employed Amber99SB-ILDN (28), one of the most widely used force fields for simulating biomolecular systems. While different improvements of RNA parameters in Amber are available (38,39), in order to be able to compare the behaviour of isolated trinucleotides with those in the context of a full tRNA, we have matched the setup of a previous study in which a full tRNA (34) was successfully simulated. Note also that most of our analysis was implemented in a self-consistent manner, whereby we have compared the relative effects for different model systems, which should potentially reduce the systematic force-field bias. This is supported by the fact that the relative carbonyl exposure of modified CCAs, as calculated from our MD trajectories, follows the experimentally observed trends when it comes to tRNA hydrolysis, especially for 3′ attachment and if split between amino-acid types.

Concerning our principal results, we have shown that 3′-aminoacylated trinucleotides exhibit a higher level of aminoacylester bond solvent exposure and also prefer more the extended conformation as compared to 2′-isomers, especially in the case of modified CCAs (Figures 1, 2, 4 and 5B). These features could make the 3′ position more susceptible to both hydrolysis and peptidyl transfer and provide a mechanistic explanation as to why for successful translation all tRNAs need to be 3′-aminoacylated, a task carried out via GTP-dependent stabilization by EF-Tu. Moreover, despite their intrinsic flexibility and structural heterogeneity, we observe a robust conformational behavior of aminoacylated trinucleotides and an invariant folding of the dominant states in MD ensembles for both positions of amino-acid attachment (Figure 4). While the nucleotide composition to a great extent defines the average preferences for the collapsed and the extended states (Figure 5B, Supplementary Figure S3A), the carbonyl solvent exposure depends on the amino-acid chemical structure, the position of its attachment and its pattern of interaction with the bases (Figure 3). Most importantly, the globally most populated collapsed conformation exhibits a significantly higher carbonyl exposure in 3′ as opposed to 2′ attachment (Figure 4E-F). Here, we have to emphasize that in the case of 2′-modified trinucleotides, it is the geometry of the attachment that enables specific base/sidechain interactions. Indeed, carbonyl exposure correlates with the MD frequency of base/sidechain interactions for both non-CCA and CCA 2′-aminoacylated trinucleotides (Figure 3B). Moreover, the same trend is seen if we compare the preference of aminoacylated trinucleotide to be in the dominant collapsed state (carbonyl protected from water) over the extended state (carbonyl exposed to water), but only in the case of 2′ attachment (Figure 4C). Thus, interaction preferences of a sidechain towards different nucleobases shape both carbonyl exposure and conformational dynamics of 2′-modified trinucleotides.

To strengthen this point, we have also compared the conformational preferences of each aminocylated trinucleotide against the knowledge-based affinities of amino acids to RNA bases (Supplementary Figure S5), previously obtained from an independent analysis of RNA-protein PDB structures (35). Note, that due to the formalism used, the positive correlations here point to the coupling between interactions and conformational preference. While the correlation for 2′-aminoacylated systems is moderate, we do not observe any reasonable correlation in the case of 3′-aminoacylated trinucleotides (Supplementary Figure S5B), in-line with the MD data discussed above. In particular, in the case of 2′ attachment, one observes a statistically significant correlation between the preference of the collapsed conformation and amino-acid affinities for the second (middle) base (*R* = 0.48, *P*-value = 0.01761, Supplementary Figure S5A), a weaker correlation for the first and a small anti-correlation for the third base (Supplementary Figure S5B). In other words, the more the sidechain prefers to interact with the middle base, the more the 2′-modified trinucleotide in question prefers to be in the collapsed, protected state than in the extended, more solvent-exposed one and *vice versa*. Interestingly, the trend for 2′-modified systems resonates with the structure of the genetic code, whereby the physicochemical character of a given amino acid is defined predominantly by the second position in its codons, with a smaller impact of the first position and the third, wobble position being largely uninformative.

These results provide an intriguing evolutionary perspective: the 2′-modified trinucleotides have a possibility to specifically recognize different amino-acid sidechains, which could be reflected in their hydrolytic stability and potentially contribute to the development of the codon/amino-acid correspondences used in translation. In this context, the dominant collapsed (folded-back) conformation of 2′-modified trinucleotides enables the nucleotides to be selective for the attached sidechain and also provides additional stability due to an additional H-bond between the 3′-hydroxyl and the phosphate oxygen of the second base. We also cannot exclude the possibility that such an H-bond-stabilized trinucleotide backbone conformation within a longer RNA molecule may provide the context for a specific recognition of the attached protein sidechain with an intriguing 3-to-1 nucleotide-to-aminoacid correspondence, which should be investigated further. It is also important to stress that only RNA molecules having both 2′- and 3′-hydroxyls on the terminal ribose, but not DNA, could develop sequence specificity to proteins based on the aforementioned structural principles.

Finally, we would like to suggest that the conformational preferences of 2′- and 3′-modified trinucleotides (or oligonucleotides in general), coupled with their differential susceptibility to hydrolysis, indeed have the potential to provide a simple, physicochemically plausible fitness criterion for the selection of certain species over others. Specifically, the ‘stereochemical hypothesis’ of the origin of the genetic code suggests that, before the ribosomal decoding and modern tRNAs arose, translation depended on direct interactions between codons and amino acids they encode (40,41). What is important here is that a specific interaction between a nucleotide triplet and a covalently bound amino acid could provide selection of some particular pairings, a process, which in turn could lead to the development of a primitive genetic code. For example, such interaction could affect the degree of protection of the aminoacylester bond from hydrolysis. Any difference in hydrolysis rates could then be amplified in multi-step reactions via kinetic proofreading (42) or similar mechanisms, leading to the selection of certain pairings between nucleotide triplets and amino acids over others. Moreover, our simulations show that the majority of 2′- or 3′-aminoacylated CCAs exhibit higher solvent exposure of the aminoacylester bond in comparison to other simulated systems (Figure 2). It is tempting to suggest that this feature, together with a lower coupling between sidechain interactions and the carbonyl exposure in the case of 3′-modification (Figure 3C), could affect the ease with which peptide synthesis occurs and may have, therefore, contributed to the choice of CCA as the universal acceptor in tRNAs. Of course, in this scenario, an additional ‘adaptor’ would be required to keep the tRNAs hydrolytically stable, a role which could be played by EF-Tu. Interestingly, for Val-modified trinucleotides we observe a segregation of L- from D-enantiomer modifications, whereby D-enantiomers exhibit the lowest aminoacylester bond solvent exposure among all simulated systems (Figure 2). Future work should show whether this feature could have influenced the choice of L-enantiomers in biological systems.

Our study provides a clear structural foundation for a plausible evolution of the RNA-protein relationship in primordial systems. At the same time, it furnishes a well-defined starting point for understanding the coupling between stereochemistry and conformational preferences in present-day tRNAs. It is precisely the stereochemical simplicity of the picture proposed herein, combined with its potentially far-reaching implications, that we hope will stimulate future experimental and computational efforts alike.

## Supplementary Material

gkz902_Supplemental_Files
