# Supplementary material for: Direct interplay between stereochemistry and conformational preferences in aminoacylated oligoribonucleotides
Source: Nucleic Acids Res. 2019 Oct 15;47(21):11077–89. doi: 10.1093/nar/gkz902 (PMC6868383; doi:10.1093/nar/gkz902)
Supplement: gkz902_Supplemental_Files [file gkz902_supplemental_files.zip › PolyanskyAA_aminoacylatedRNA_manuscript_NAR_Supplementary_Data_revised25072019.pdf]

Supplementary Data

Direct interplay between stereochemistry and conformational preferences in aminoacylated oligoribonucleotides

Anton A. Polyansky[1,2]*, Mathias Kreuter[1], John D. Sutherland[3], Bojan Zagrovic[1]*

[1]Department of Structural and Computational Biology, Max Perutz Labs, University of Vienna, Campus Vienna Biocenter 5, Vienna A-1030, Austria
[2]Higher School of Economics, Myasnitskaya 20, 101000 Moscow, Russia
[3]MRC Laboratory of Molecular Biology, Francis Crick Avenue, Cambridge Biomedical Campus, Cambridge CB2 0QH, UK

*To whom correspondence should be addressed. Tel: +43 1 4277 52271; Fax: +43 1 4277 9522; Email: bojan.zagrovic@univie.ac.at. Correspondence may also be addressed to newant@gmail.com.

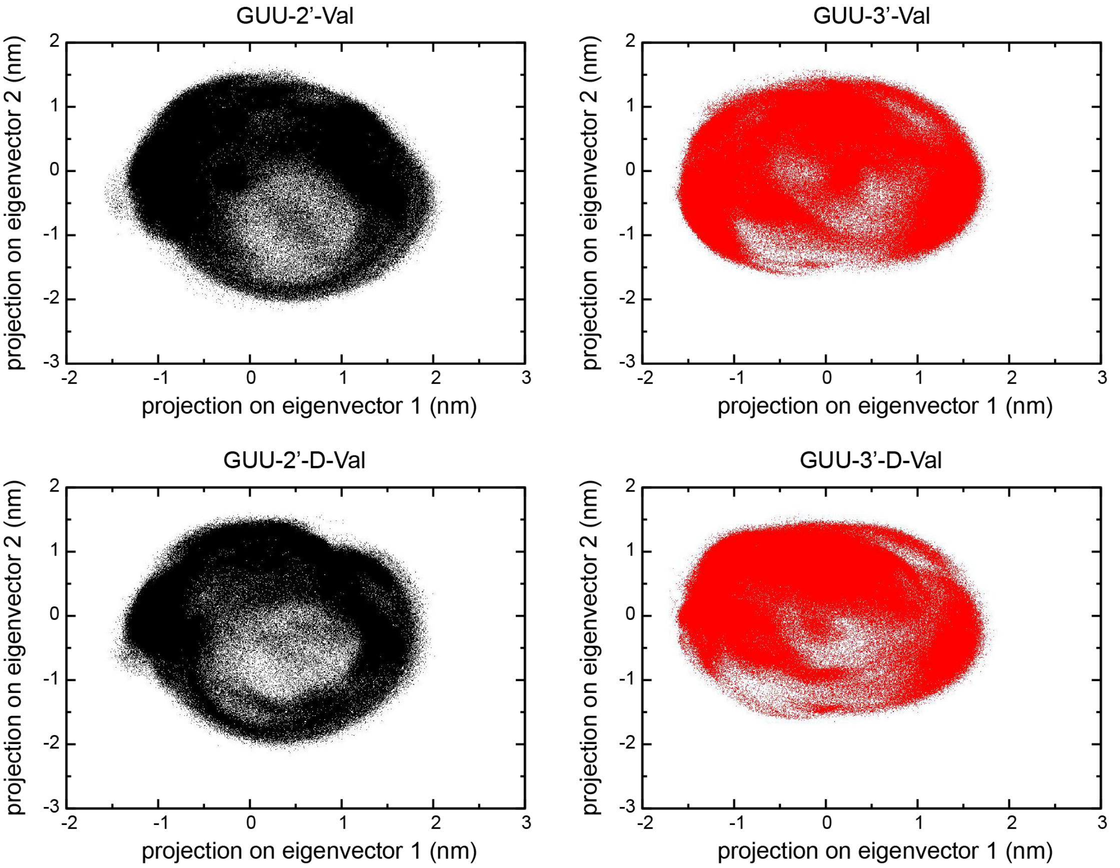

Supplementary Figure S1. Available conformational space of aminoacylated trinucleotides. Projection of MD trajectories of different GUU-Val stereoisomers onto the first and the second eigenvectors from the PCA analysis of backbone conformations of aminoacylated trinucleotides from the joint MD trajectories.

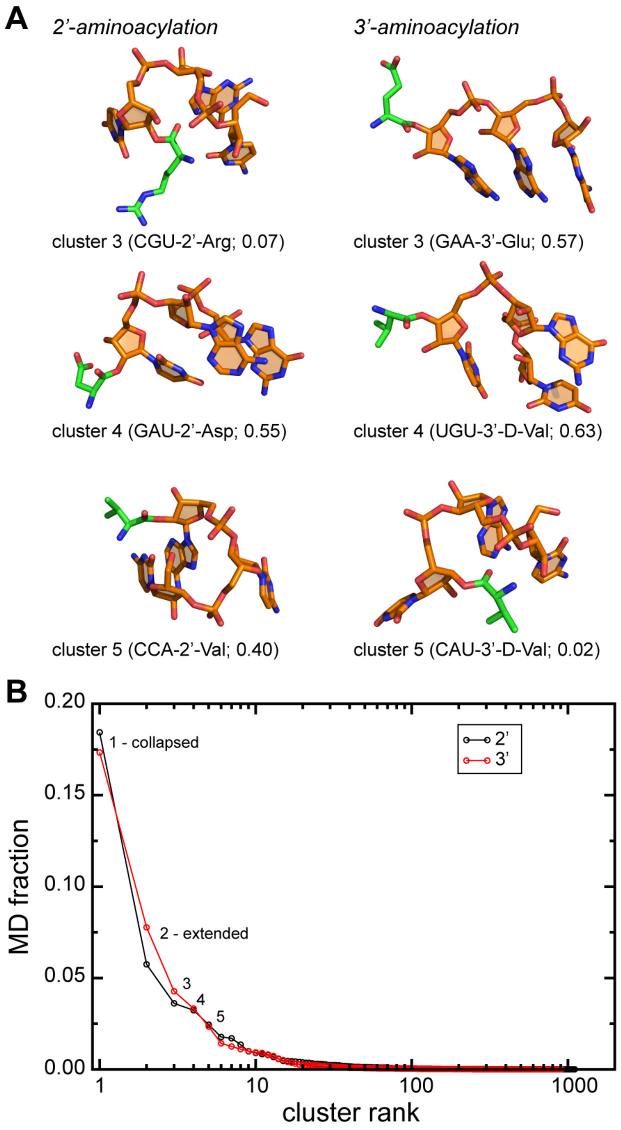

Supplementary Figure S2. Analysis of structural clusters of aminoacylated trinucleotide backbone conformations in the case of 2’- and 3’- attachment. A) Representative conformations for top 5 most populated structure clusters (ranks 3-5) For the dominant states (ranks 1-2), please check Figure 3A-B in the manuscript. Cluster centers are given in stick representation. B) Distribution of cluster populations as a function of cluster rank. The *x*-axis is given on a log scale.

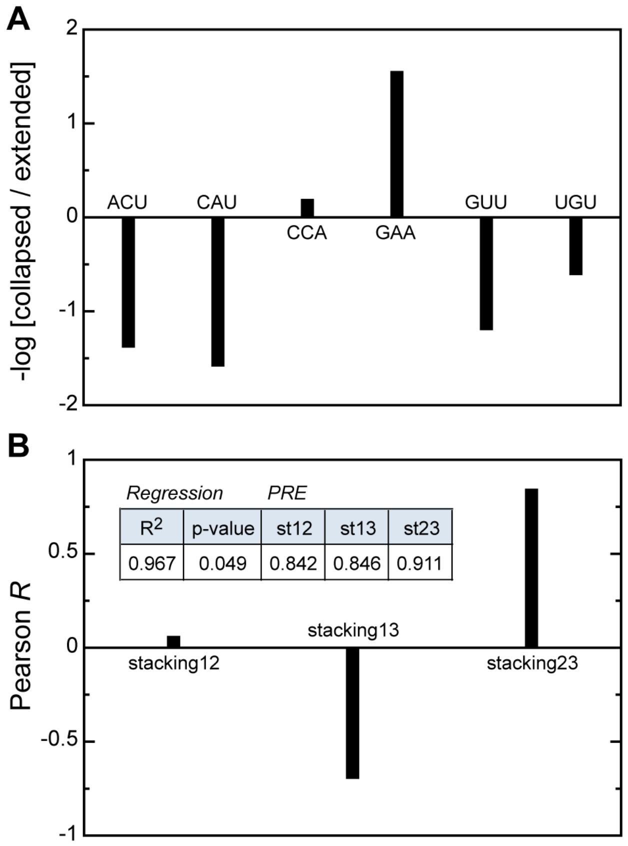

Supplementary Figure S3. Composition of trinucleotides defines their intrinsic conformational preferences. A) Trinucleotide conformational preference for the *collapsed* over the *extended* state. B) Pearson correlation coefficients *R* between the stacking interaction frequencies of different pairs of bases and the conformational preference for the *collapsed* over the *extended* state. The inset displays the results of the proportional reduction in error (PRE) treatment of the multiple regression analysis results probing the relationship between the relative preferences of collapsed and extended states and set of stacking interactions used as predictors. The uniformly high PRE values indicate that each of the three predictors contribute substantially to the quality of the regression.

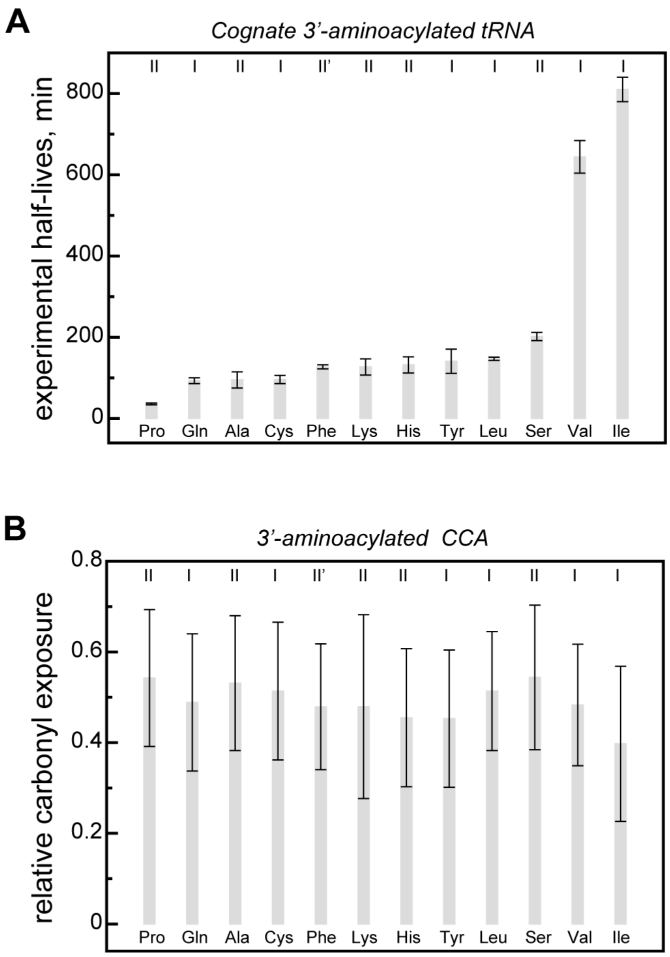

Supplementary Figure S4. Carbonyl exposure in MD simulation captures experimental trends. A) Hydrolytic stability of aminoacylated yeast tRNAs (cognate pairs, data from Peackock et al. (21)). Aminoacyl-tRNA synthetase classes are indicated with the Roman numerals on the top. B) Relative average carbonyl solvent exposure for the corresponding 3'-aminoacylated CCAs.

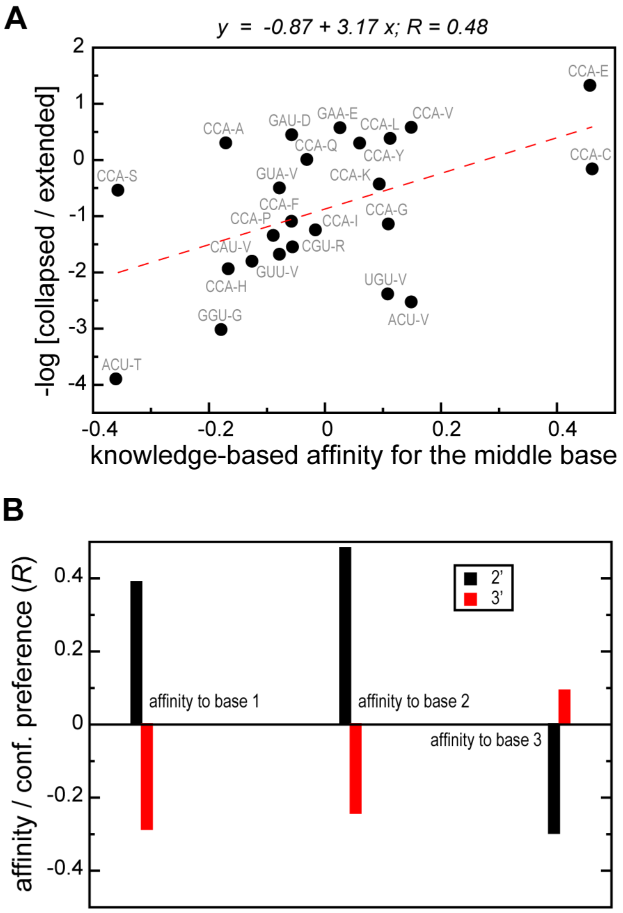

Supplementary Figure S5. Specificity of the sidechain for trinucleotide bases tunes the conformational preference of collapsed over extended states. A) Linear regression between the knowledge-based affinity of the sidechain from Polyansky et al. (35) for the middle base in 2'-aminoacylated nucleotides and the conformational preference for the *collapsed* over the *extended* state. B) Pearson correlation coefficients R between the knowledge-based affinities to different bases and the conformational preference for the *collapsed* over the *extended* state. The black and red bars correspond to 2'- and 3'-aminoacylated trinucleotides, respectively.

Supplementary Table S1. MD derived parameters for each simulated system.

Uploaded as dataset

Supplementary Table S2. Ranking of the three dominant conformational clusters in each individual MD trajectory.

Uploaded as dataset

Supplementary Table S3. Carbonyl exposure and conformational preference described as linear models involving key intramolecular interactions in aminoacylated trinucleotides.

Dataset for analysis			Multiple linear regression		PRE* for predictors					
attachement	trinucleotides	response variable	R^2	p-value	b1	b2	b3	st12	st13	st23
2'	all	carbonyl exposure	0.5761	0.0023	0.0674	0.0908	0.0435	0.0026	0.0119	0.1706
2'	CCA	carbonyl exposure	0.5965	0.2457	0.0054	0.2432	0.2561	0.0002	0.0421	0.2466
2'	non CCA	carbonyl exposure	0.8137	0.0131	0.0145	0.0263	0.0408	0.1849	0.3232	0.5138
3'	all	carbonyl exposure	0.5457	0.0216	0.0702	0.112	0.1463	0.1686	0.0001	0.0095
3'	CCA	carbonyl exposure	0.2991	0.7932	0.1769	0.0577	0.2367	0.0775	0.0058	0.0012
3'	non CCA	carbonyl exposure	0.748	0.4012	0.1384	0.0041	0.0003	0.0931	0.0533	0
2'	all	-log[collapsed/extended]	0.677	0.0002	0.2755	0.3435	0.2485	0.4652	0.3146	0.1392
2'	CCA	-log[collapsed/extended]	0.6561	0.1594	0.169	0.3643	0.3322	0.0385	0.0382	0.1917
2'	non CCA	-log[collapsed/extended]	0.8119	0.0136	0.3272	0.5409	0.3248	0.7027	0.645	0.3169
3'	all	-log[collapsed/extended]	0.1239	0.8683	0.0024	0.075	0.0006	0.0038	0.0086	0.0043
3'	CCA	-log[collapsed/extended]	0.763	0.0535	0.5897	0.1049	0.6398	0.6015	0.0024	0.3737
3'	non CCA	-log[collapsed/extended]	0.7579	0.3828	0.3768	0.1625	0.4459	0.006	0.1399	0.3555

*PRE, Proportional Reduction in Error (see Methods for details): b1, b2, and b3 – sidechain interactions with base 1, base 2, and base 3, respectively; st12, st13, and st23 – stacking interactions between corresponding bases. The significance of different values is indicated in shades of green (dark green – most significant).

Supplementary Table S4. Carbonyl exposure and conformational preference described as linear models of key intramolecular interactions in aminoacylated CCAs.

Dataset for analysis			Multiple linear regression		PRE* for predictors					
attachement	trinucleotides	response variable	R^2	p-value	b1	b2	b3	st12	st13	st23
2'	CCA	carbonyl exposure	0.4177	0.1297	0.0283	0.3108	0.2487			
			0.2809	0.3272				0.0732	0.1086	0.2245
2'	CCA class I	carbonyl exposure	0.9193	0.038	0.491	0.2005	0.8557			
			0.7903	0.1524				0.6668	0.4499	0.6769
2'	CCA class II	carbonyl exposure	0.7775	0.1658	0.3828	0.6654	0.7072			
			0.5649	0.4176				0.1051	0.4209	0.5433
3'	CCA	carbonyl exposure	0.1537	0.6264	0.0249	0.0164	0.1241			
			0.0276	0.9613				0.0137	0.0197	0.0112
3'	CCA class I	carbonyl exposure	0.1878	0.8699	0.1457	0.0877	0.0519			
			0.5148	0.4811				0	0.053	0.0954
3'	CCA class II	carbonyl exposure	0.6934	0.26	0.2097	0.0433	0.6626			
			0.5052	0.4934				0.3933	0.4096	0.1136
2'	CCA	-log[collapsed/extended]	0.5478	0.0403	0.1458	0.2337	0.4698			
			0.1387	0.6676				0.002	0.0203	0.1224
2'	CCA class I	-log[collapsed/extended]	0.969	0.0092	0.8907	0.9524	0.9657			
			0.5898	0.3863				0.1014	0.4602	0.4952
2'	CCA class II	-log[collapsed/extended]	0.6949	0.2583	0.2535	0.1701	0.6252			
			0.3317	0.7101				0.292	0.2955	0.0981
3'	CCA	-log[collapsed/extended]	0.1403	0.6631	0	0.0024	0.1262			
			0.2321	0.4293				0.0322	0.076	0.0419
3'	CCA class I	-log[collapsed/extended]	0.474	0.5331	0.2374	0.0803	0.3453			
			0.373	0.66				0.0713	0.0054	0.189
3'	CCA class II	-log[collapsed/extended]	0.1457	0.9098	0.0123	0.0056	0.1256			
			0.7965	0.146				0.1868	0.6584	0.5154

*PRE, Proportional Reduction in Error (see Methods for details): b1, b2, and b3 – sidechain interactions with base 1, base 2, and base 3, respectively; st12, st13, and st23 – stacking interactions between corresponding bases. Due to the limited number of data points in each CCA subset grouped according to the corresponding aminoacyl tRNA-synthetase class (7 points in each subset), the multiple regression analysis was performed using as predictors sidechain-base interactions or, separately, stacking interactions only. The significance of different values is indicated in shades of green (dark green – most significant).
